# Sulfide Oxidation Products Support Microbial Metabolism at Interface Environments in a Marine‐Like Serpentinizing Spring in Northern California

**DOI:** 10.1111/gbi.70026

**Published:** 2025-06-25

**Authors:** Leah Trutschel, Brittany Kruger, Andrew Czaja, Megan Brueck, Joshua Sackett, Gregory Druschel, Annette Rowe

**Affiliations:** ^1^ Department of Biological Sciences University of Cincinnati Cincinnati Ohio USA; ^2^ Division of Hydrologic Sciences Desert Research Institute Las Vegas Nevada USA; ^3^ Department of Geosciences University of Cincinnati Cincinnati Ohio USA; ^4^ Yale College Yale University New Haven Connecticut USA; ^5^ Department of Microbiology, Genetics and Immunology Michigan State University East Lansing Michigan USA; ^6^ Department of Earth Sciences Indiana University Purdue University Indianapolis Indianapolis Indiana USA

**Keywords:** alkaliphiles, polysulfide, Serpentinization, sulfur oxidation, thiosulfate

## Abstract

Interface environments between extreme and neutrophilic conditions are often hotspots of metabolic activity and taxonomic diversity. In serpentinizing systems, the mixing of high pH fluids with meteoric water, and/or the exposure of these fluids to the atmosphere can create interface environments with distinct but related metabolic activities and species. Investigating these systems can provide insights into the factors that stimulate microbial growth, and/or what attributes may be limiting microbial physiologies in native serpentinized fluids. To this aim, changes in geochemistry and microbial communities were investigated for different interface environments at Ney Springs—a marine‐like terrestrial serpentinization system where the main serpentinized fluids have been well characterized geochemically and microbially. We found that reduced sulfur species from Ney Springs had large impacts on the community changes observed at interface environments. Oxygen availability at outflow environments resulted in a relative increase in the taxa observed that were capable of sulfur oxidation, and in some cases light‐driven sulfur oxidation. A combination of cultivation work and metagenomics suggests these groups seem to predominantly target sulfur intermediates like polysulfide, elemental sulfur, and thiosulfate as electron donors, which are present and abundant to various degrees throughout the Ney system. Fluid mixing with meteoric water results in more neutral pH systems which in turn select for different sulfur‐oxidizing taxa. Specifically, we see blooms of taxa that are not typically observed in the primary Ney fluids, such as *Halothiobacillus* in zones where fluids mix underground with meteoric water (~pH 10) or the introduction of *Thiothrix* into the nearby creek as fluids enter at the surface (~pH 8). This work points to the potential importance of oxidants for stimulating microbial respiration at Ney Springs, and the observation that these serpentinized fluids act as an important source of reduced sulfur, supporting diverse taxa around the Ney Springs system.

## 1 Introduction

In an ecological context, interface environments are formed where two systems of distinct conditions merge and exchange energy and resources (Mihailović et al. 2017). Due to this mixing, these boundary zones are often hotspots of metabolic activity and taxonomic diversity. In astrobiology, interfaces are of particular interest, as they are thought to have played a key role in the origin of life on our planet, and/or play a role in supporting life from geologically driven redox gradients (Boyd et al. 2020; Martin et al. 2008; Russell et al. 2010). In addition, these settings provide an opportunity to investigate the conditions that may be limiting microbial growth and/or how population structure and activity change across gradients of different environmental conditions. A notable example of the importance of interfaces includes outflows of Yellowstone hot springs, which have provided insight into the temperature extremes at which different auto/phototrophic communities can survive and/or perform optimally (Boomer et al. 2009; Hamilton and Havig 2022). Similarly, pH is an important environmental parameter that can include or exclude microbial members and specific physiologies, though work across pH gradients, especially in alkaline systems, is limited. This work investigates the geochemistry and geomicrobiology of naturally occurring redox and pH gradients that form at interfaces of high conductivity serpentinized fluids mixing with surface/meteoric fluids or atmospheric (i.e., oxic) gases.

Serpentinizing systems arise from a geologic process in which water hydrates ultramafic rock resulting in the production of high pH fluids (> pH 10) rich in hydrogen, and occasionally methane or other small organic compounds (McCollom and Seewald 2013; Schrenk et al. 2013). As a result, these reduced fluids are often enriched in electron donors but lack oxidants to support microbial respiration. Thus, mixing with oxidant‐rich fluids or the atmosphere can provide an important resource for microbial life. Additionally, this mixing can ameliorate some of the extreme conditions (i.e., decrease pH) that limit habitability in the reacted fluids. Examples of life capitalizing on these interface‐based niches can be observed in the Lost City serpentinizing system, where hydrothermal fluids rich in hydrogen mingle with sulfate and oxygen rich seawater; this merging supports a dynamic microbial food web including hydrogen oxidizers, methano‐gen/‐trophs, and sulfur‐reducers/‐oxidizers (Kelley et al. 2001; Spear and Pace 2004). In terrestrial systems, exposure of reduced serpentinized fluids to either the atmosphere or more oxidized fluids found in springs formed at the surface can also generate additional oxidants. These geochemical interactions also play an important role in shaping the microbial community. Consequently, surface springs are often dominated by oxygen consuming hydrogenotrophs such as *Serpentinamonas* (Cedars/Tablelands etc.) (Brazelton et al. 2012; Suzuki et al. 2014), On the other hand, the limited concentration of sulfate/sulfide in terrestrial fluids compared to marine environments often limits the abundance of sulfur‐reducing/oxidizing taxa found within these systems (Brazelton et al. 2006; Suzuki et al. 2017; Woycheese et al. 2015). In the few terrestrial systems that do harbor sulfur‐associated taxa, such as the Coast Range Ophiolite Microbial Observatory and the Samail ophiolite, geologically derived sulfur from ancient marine deposits is the proposed source (Glombitza et al. 2021; Sabuda et al. 2020). Overall, the variation in host geology, the influence of meteoric water and the depth sampled can result in extensive geochemical and microbial community variation even within the same systems, such as is seen in The Cedars (Morrill et al. 2013; Suzuki et al. 2013), Manleluag (Woycheese et al. 2015), and the Samail ophiolite (Rempfert et al. 2017). Comparing microbial communities across the gradients present in these systems could provide insight into how core microbial taxa respond positively and negatively to the various geochemical changes encountered. Thus, this type of study will provide further insight into the ecology of these systems, supporting which taxa are positively or negatively influenced by environmental changes, and/or which chemical species play important roles as limiting or stimulating nutrients.

**FIGURE 1:**
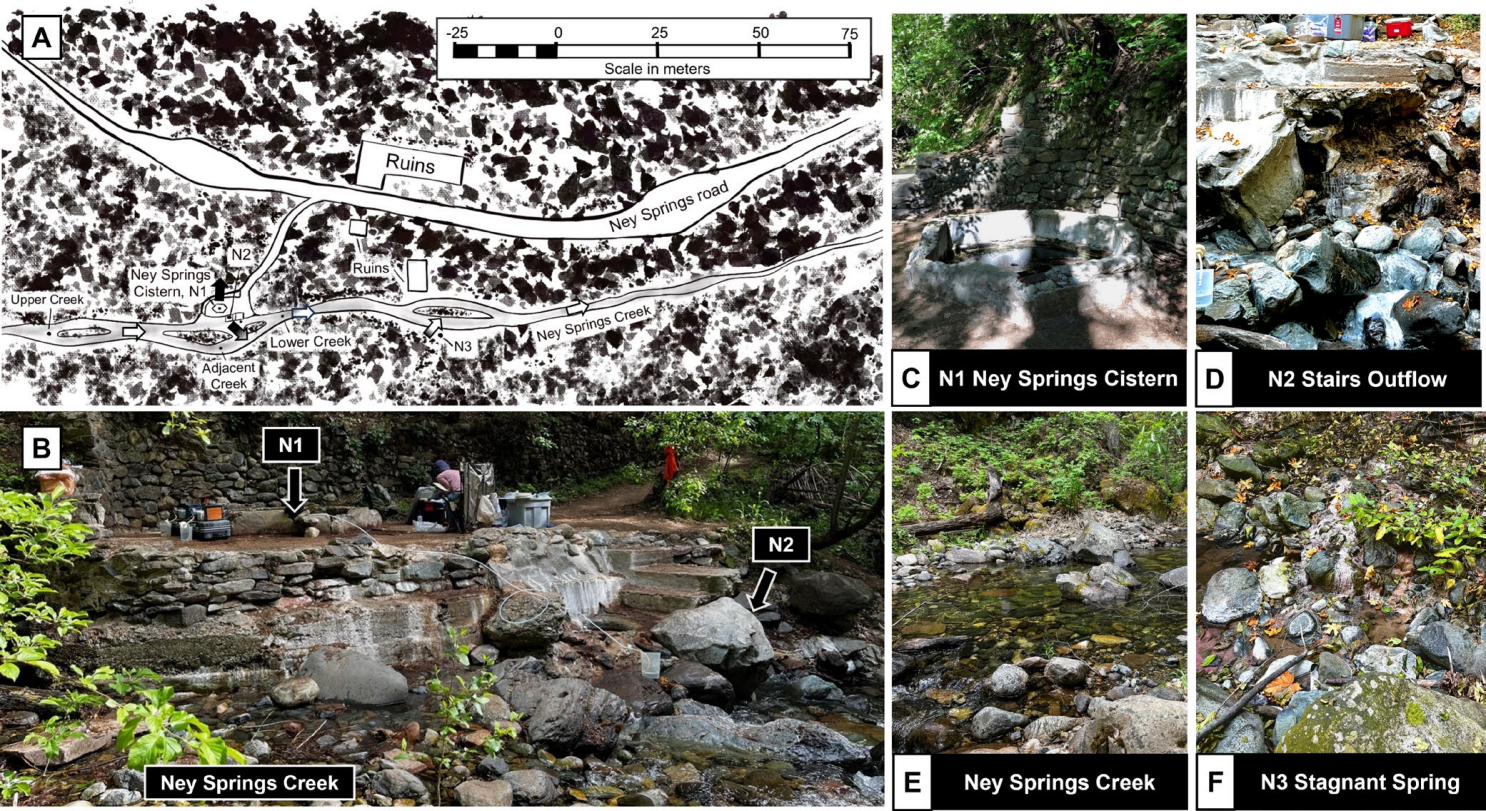
(A) Redrawn map of Ney Springs area from Feth et al. (1961). The small arrows indicate approximate fluid composition and flow direction. Meteoric water is represented by white, will serpentized fluids are represented by black. Ney Springs Creek is primarily meteoric water that flows downward to the east. The cistern (**N1**) is primarily serpentinized fluids that upwell from cracks in the bottom of the structure, as indicate by a black arrow pointed up. The stairs outflow (**N2**) is primarily serpentinized fluids with some meteoric water introduced as it drains into Ney Springs Creek, while Stagnant Spring (**N3**) is primarily meteoric water with slight underground mixing with serpentinized fluids that trickles down from the embankment into Ney Springs Creek. (B) View of Ney Springs cistern and stairs outflow from standing at adjacent Ney Springs Creek point. (C) **N1** is the main cistern as viewed from coming down the trail. (D) Below the stairs lies the **N2** stairs outflow which is frequently characterized by the presence of a biofilm on the vertical stone surfaces. (E) View of Ney Springs creek as seen at the lower creek location designated on the map. (F) **N3** is a view of Stagnant Spring as seen when standing in front of the resort ruins in Ney Springs Creek.

Ney Springs is a small spring located in Northern California and has previously been shown to be distinct from other terrestrial serpentinizing systems both geochemically and microbially (Trutschel et al. 2022). It has marine‐like geochemistry with high conductivity fluids (35 mS/cm) that are 10× higher than the previously highest reported measurement for a terrestrial system (i.e., 3 mS/cm measured at the Tablelands) (Trutschel et al. 2022, 2023). The spring has high pH fluids (12–12.5) that are rich in ammonia (> 100 mg/L), methane (17 mg/L dissolved), and sulfide (> 700 mg/L) (Trutschel et al. 2022). The fluid geochemistry of Ney Springs is likely influenced by the Franciscan subduction complex (marine deposit) and/or regional basaltic formations, as evidenced by Ney fluids being highly conductive and containing elevated levels of sodium (15,000 mg/L) and silica (4000 mg/L) (Feth et al. 1961; García‐Ruiz et al. 2017; Trutschel et al. 2023). The source of Ney Springs fluids primarily collect into a concrete cistern constructed ca. 1889 (Figure 1), and previous geochemical and microbial community characterization of this system have focused on the fluids contained in this fixture (Cook et al. 2021; Feth et al. 1961; Trutschel et al. 2022). Repeated isotopic measurements support that the fluids observed in the main cistern at Ney Springs, are connate in origin, as they fall well off the meteoric water line and show a consistently depleted δ^18^O isotopic composition (Figure S1). In addition to the primary cistern structure, however, there are several outflow seepage spots where high pH fluids can be observed draining into a proximal creek. Downstream, there is also stagnant spring, which is much lower in pH, though still sulfidic and predicted to be the product of serpentinized and meteoric fluid mixing (Feth et al. 1961). In this work we investigate how serpentinized fluids exposed to and mixing with the surrounding environment affects microbial community composition and changes in geochemistry, with a particular focus on sulfur, to further investigate the importance of different environmental parameters for supporting or promoting microbial life in these geologically altered fluids.

## 2 Materials and Methods

### 2.1 Site Description

The physical and geological setting of Ney Springs has been previously described (Feth et al. 1961), overview in Figure 1A,B. The main regions of access to serpentinized fluids in the area are described as follows. The cistern consists of an octagonal concrete structure approximately 1 m wide and 1 m deep (**N1**) (Figure 1C). Fluids seep into the cistern from the bottom, where methane rich gases are also continuously bubbling into the system. The cistern has previously been emptied and fluids replenished the system through cracks in the bottom of the structure (Feth et al. 1961). We estimate, based on the rate of replenishment of removed fluids, that the fluid flow rate is approximately 4 L/h. Loss of fluid occurs through cracks in the side walls of the cistern and a metal pipe (visible from the side of the cistern) from which fluids slowly drain into the surrounding area. Fluids from the cistern drain to the stairs outflow (**N2**, Figure 1D), which is where fluid can be seen seeping out of rocks beneath the concrete stairs leading down to the creek In warmer months, a biofilm community can often be observed where these fluids pass over the substrate. These fluid samples were collected to assess aqueous geochemistry of the stairs outflow environment, and biomass from the biofilm communities in contact with the fluids were used to profile microorganisms. A notable feature of Ney Springs creek is that cobbles within the stream are covered in white filamentous biofilms just downstream of the stairs outflow area (Figure 1E). Further downstream is stagnant spring (**N3**, Figure 1F), a small cascade located along the bank approximately 70 m down from the main cistern. It features heavy pink and white filamentous biofilm growth where the fluids run down the bank into Ney Springs creek. Three other locations, “Lodge Spring”, “Sweet Spring”, and “Sulfur Spring” were noted previously (Feth et al. 1961), but could no longer be located.

### 2.2 Geochemical Sampling

The Ney Springs primary cistern and surrounding points of interest were sampled between May 2021 and May 2023. Conductivity, pH, total dissolved solids (TDS) and oxidation–reduction potential (ORP) were measured using a multimeter (Columbus OH, USA). Sulfide and dissolved oxygen were measured with a HACH portable spectrometer using methods 8316 and 8131 (Loveland, CO, USA). Thiosulfate and tetrathionate were also measured with the HACH utilizing the cyanolysis method (Kelly et al. 1969). Cistern and creek fluids were collected using a Geopump peristaltic pump (GeoTech, Denver, CO, USA) and autoclaved MasterFlex PharMed BPT tubing (Cole‐Palmer, Vernon Hills, IL, USA) equipped with a polypropylene in‐line filter housing (Millipore; Bedford, MA, USA) containing 0.1 μm polycarbonate membrane filters (47 mm diameter, Millipore, Tullagreen, Carrigtwohill Co. Cork, IRL). Outflow and stagnant spring fluid samples were collected using syringes fitted with 0.1 μm syringe filters. Samples for cation (Li^+^, Na^+^, NH_4_^+^, K^+^, Mg^2+^, and Ca^2+^) and anion (F^−^, Cl^−^, NO_2_^−^, Br−, NO_3_^−^, PO_4_^3−^ and SO_4_^2−^) analysis were measured on a Dionex Aquion Ion Chromatograph (Thermo Fisher Scientific, Waltham, MA, USA). Technical replicates of standards and water samples were performed with standard errors observed at less than 2% for each analyte. Creek and stagnant spring samples were analyzed undiluted, while the cistern, footbath, and outflow samples were run at a 1:10 dilution with MilliQ water, or at a 1:5 dilution with MilliQ water after the sample had been mixed with Amberlite MB20 H/OH resin beads (Sigma‐Aldrich, USA, with a ratio of 80 mg of beads per 2 mL of sample). The removal of chloride with the resin beads allowed for better detection of less abundant species such as nitrate and nitrite.

### 2.3 Isotope Analysis

Water isotope samples were collected and analyzed at the Center for Stable Isotope Biogeochemistry at the University of California, Berkeley using Isotope Ratio Mass Spectrometry (IRMS) as described previously in (Trutschel et al. 2023). This method maintains a long‐term external precision for δ^2^H and δ^18^O of ±0.6^0^/_00_ and ± 0.12^0^/_00_ respectively. Samples for sulfide isotopes were precipitated as ZnS by mixing Ney water with 0.5 M Zinc Acetate (pH 4.0) in a 1:1 ratio. Sulfate was precipitated with 5 mL of 1 M BaCl maintained at pH 4.0 using HCl (Canfield et al. 2010). In both cases precipitates were filtered in the field onto glass fiber filters (GFF) and stored in sterile conical tubes until analysis. Triplicate samples were preserved. Isotope analysis was performed at the Geobiology laboratory at MIT as previously described, with the reported analytical standard deviations are ±0.4, 0.02, and 0.16 for δ^34^S, Δ^33^S and Δ^36^S, respectively. (Uveges et al. 2023). In brief, ZnS was converted to Ag_2_S using AgNO_3_. Ag_2_S was then converted to SF_6_ which was cryogenically purified in liquid nitrogen and gas‐source isotope ratio mass spectrometry was performed using a Thermo‐Finnigan MAT 253 (Thermo Scientific). Sulfate from the BaSO_4_ precipitate was reduced to sulfide using Thode chemistry (Farquhar 2008) and subsequently analyzed with the SF_6_ method.

Continuously exsolving gas bubbles were captured via an inverted funnel inserted into a submerged and inverted 150 mL serum bottle, and were used to analyze ^13^C and ^2^H in bulk methane gas at the University of California, Davis Stable Isotope Facility (UCD‐SIF) via concentration (ThermoScientific Precon, Bremen, Germany) and subsequent IRMS analysis (ThermoScientific Delta V Plus, Bremen, Germany) following standard UCD‐SIF procedures. Resulting δ^13^C and δ^2^H of methane are reported relative to international standards (V‐PDB (Vienna PeeDee Belemnite) for carbon and V‐SMOW (Vienna‐Standard Mean Ocean Water) for hydrogen). The long‐term standard deviation for methane analysis via GasBench‐Precon‐IRMS reported is ±0.2^0^/_00_ and ± 2^0^/_00_ for δ^13^C‐CH_4_ and δ^2^H‐CH_4_ respectively.

### 2.4 Sulfur Electrochemistry

Sulfur and oxygen species presence within the cistern and outflow communities was assessed using cyclic voltammetry (CV) with gold‐amalgam microelectrodes as previously described (Brendel and Luther 1995; Luther et al. 2000, 2008). Au microelectrodes were purchased from Analytical Instrument Systems Inc. (AIS) (http://02f7766.netsolhost.com/, New Jersey, USA) and plated in house to form Au‐Hg amalgam electrodes according to (Luther et al. 2008). A three‐electrode system was used consisting of the Au‐Hg working electrode, Ag/AgCl reference electrode, and platinum wire counter electrode (Figure S2). CV scans were performed between −0.1 and −2.0 V (vs. Ag/AgCl) at a scan rate of 1000 mV/s using an AIS DLK Micro‐Pstat‐1 portable potentiostat. CVs were run in batches of 10 with the final three scans used for confirmation of presence/detection rather than quantification. Approximate location of sulfur species peaks were referenced from (Druschel et al. 2003; Luther et al. 2008; Rosen 1971) or were concluded experimentally by adding known concentrations of sulfide and/or polysulfide (S_4_) to an anoxic Ney Springs artificial medium consisting of 200 mM NaCl,10 mM NH_4_Cl, 0.5 mM MgCl_2_* 6H_2_O, 1 mM K_2_HPO_4_, 100 mM Na_2_CO_3_, and 5 mM NaOH adjusted to pH 12. Polysulfide (S_4_) was prepared according to (Boyd and Druschel 2013). Electrode conditioning was performed in between additions of standards or dilutions in the field, with the electrode placed in MilliQ water and poised at −1.4 V for 1 min to remove any residual sulfur species from the working electrode. After re‐conditioning, the electrodes were then transferred to a new vessel with fresh matrix and a CV ran again to ensure removal of all remaining sulfur species before proceeding with more testing. Ney cistern fluids were added to a vial of anaerobic MilliQ water to perform dilutions. This was given a brief stir and then electrodes were immediately added to limit oxygen intrusion. Electrode tests on the **N2** outflow area were conducted by placing the electrodes directly into the biofilm or within small pools of liquid (Figure S2).

### 2.5 Sulfur Spectroscopy

Fluid samples for UV–visible spectrophotometry were collected from **N1**, **N2**, and **N3**. These were analyzed in 1 cm cuvettes in a Genesys 150 spectrophotometer (Thermo Fisher Scientific, WI, USA). Absorbance was measured over wavelengths from 200 and 1100 nm to encompass the polysulfide range (Khan 2012). Wavelength accuracy is reported at ±0.5 nm with repeatability at < ±0.2 nm. Data was collected using a medium speed scan rate at 1 nm increments. Photometric accuracy for this instrument is reported at ±0.002A at 0.5A, ±0.004A at 1.0A, ±0.008 A at 2.0 A, with photometric reproducibility at ±0.001 A at 1 A measured at 1.0 A at 546 nm.

Fluid and mineral precipitate samples from **N1** and **N2** were collected and frozen for Raman analysis on a Horiba MacroRAM Raman spectrometer, which uses a 785 nm excitation laser. The laser was operated at 100% power (for water samples) or 40% power (for precipitate) and spectra were collected for 10 to 30 s. Fluid samples were analyzed in a fused quartz cuvette, and baseline corrected using data from the blank cuvette. Precipitate samples were pressed into a sample holder for analysis. Spectra were compared to the RUFF elemental sulfur standard (https://rruff.info; **RRUFF ID**: R050006).

### 2.6 16S rRNA Sample Collection and Analysis

Filtered fluids and solid material (biofilms) were collected from sites **N1–N3** and the adjacent creek across eight sampling trips. Filtered fluid samples were collected from the cistern and creek using the peristaltic pump and in‐line filter housing setup described in the geochemical sampling section, with different sets of tubing and filter housing used for each location. Preservation of the cistern filters was described previously, with filtering continuing until clogging (falling between 2 and 12 L) (Trutschel et al. 2023). A similar collection method of creek filters was performed with the exception that only 2 L of fluid was filtered for each sample. Biofilm/sediment samples from the **N2** and **N3** locations were collected with autoclaved scopulas into sterile 15 mL conical tubes and preserved on dry ice then at −20°C until DNA extraction. This included both solid materials and fluids from each location. DNA was extracted from filters or collected biomass using a Qiagen DNAeasy Powersoil kit and quantified using a Qubit fluorometer (ThermoFisher Scientific, USA).

The V4 region 16S rRNA region (515F‐806R) was sequenced via Novogene (en.novogene.com) (Beijing, CHN) using NovaSeq PE250 amplicon sequencing. A total of 39 samples were used for overall community analysis, with the cistern fluid samples (**N1**) previously analyzed (Trutschel et al. 2023). Here we additionally include 12 biofilm samples which were obtained from the stairs outflow (**N2**), two biofilm samples which were obtained from Stagnant Spring (**N3**), and four fluid samples obtained from three locations along the Ney Springs Creek spanning upstream and downstream of the main cistern location. All 16S data can be found at NCBI under accession number SRP324941. Sequence data was processed using DADA2 (v. 1.22.0) (Callahan et al. 2016) for quality filtering. DeContam (v. 1.14.0) (Davis et al. 2018) was used for erroneous sequence removal using the prevalence method, which takes into account presence/absence of amplicon sequence variants (ASVs) in control vs. true samples and removes them from the true samples. The extraction blank used as the control sample contained very few shared sequences with most of the environmental samples, so a threshold of 0.375 was set to remove contaminants that were most abundant in the blank. The list of sequences flagged as contaminants was then manually reviewed to prevent accidental removal of true environmental sequences. Chloroplast and mitochondrial sequences were filtered out prior to analysis. Phyloseq (v. 1.38.0) (McMurdie and Holmes 2013) was used for taxonomic bar charts and Bray‐Curtis distance calculation, while Dendextend (v. 1.16.0) (Galili 2015) was used for plotting the Bray‐Curtis dendrogram. Differential abundances of sequences compared between samples was determined using DESeq2 (Love et al. 2014). To focus on taxa found throughout the system and less likely to be introduced from the surroundings, only taxa found in five or more samples and with a total count greater or equal to 500 were included for comparisons in the DEseq2 analysis. This filtering is due to findings from our previous work that highlighted a high transient population of ASVs that were not found in all our sampling events and were generally in abundances < 1% (Trutschel et al. 2023). These cut‐offs were instituted to limit observations in these transient populations in the results.

### 2.7 Metagenome Analysis

Metagenome assembled genomes (MAGs) from the Ney Spring's interface environments were compiled and/or compared with Ney Springs cistern metagenome data, using the sampling, DNA extraction, and sequencing methods previously described in (Trutschel et al. 2022) and (Trutschel et al. 2023). For clarity, the origin of each MAG is indicated in Table S1. Sequenced MAGs were analyzed in the Kbase web platform (Arkin et al. 2018) according to the metagenome analysis method used in (Trutschel et al. 2023). Briefly, the metagenomic reads were co‐assembled by IDBA‐ID (v1.1.3) (Peng et al. 2012), MEGAHIT (v1.2.9) (Li et al. 2015), and metaSPAdes (v 3.15.3) (Nurk et al. 2017). From this, CONCOCT (v1.1) (Alneberg et al. 2014), MaxBin2 (V2.2.4) (Wu et al. 2016) and MetaBAT2 (v1.7) (Kang et al. 2019) were used to generate bins, with DAS tool (v1.1.2) (Sieber et al. 2018) then used to combine redundant bins generated across all three binning methods into one binned set for each of the three assembly variants. The three assemblies were then classified using GTDB‐tk (v1.7.0) (Chaumeil et al. 2020) and assessed in CheckM (v1.0.18) (Parks et al. 2015) for completeness. The multiple sequence alignment CheckM creates to assess bin completeness was then used to generate a phylogenetic tree using FastTree2 (v2.1.9) (Price et al. 2010). As many of the bins were represented in each of the three assembly methods, the phylogenetic tree and CheckM stats were then used to choose which bin would be representative based on having the highest completeness and lowest contamination. Unique bins only generated for one of the assembly methods were also evaluated. From this method, a final list of MAGs was obtained. These MAGs were then annotated using KEGG GhostKoala (v2.2.) (Kanehisa et al. 2016). From this large pool of MAGs, genomes that contained genes related to sulfur metabolism were screened. Metabolic pathway completeness for sulfur oxidation, disproportionation, and reduction was assessed using the KeggDecoder package (Graham et al. 2018) and by manual search through the KEGG annotations. Metagenome data is available under the NCBI BioProject accession number PRJNA739719.

### 2.8 Bioorthogonal Noncanonical Amino Acid Tagging (BONCAT) Setup and Analysis

BONCAT was used to determine the activity of microorganisms within Ney Springs cistern fluids when enriched with different sulfur species. Vials and analyses were all prepared using the BONCAT protocol, with homopropargylglycine (HPG) used as the methionine analog as it is recommended for high pH and sulfide rich conditions (Hatzenpichler and Orphan 2015). 170 mL serum vials were purged with N_2_ and then crimped. An HPG stock solution was prepared at a concentration of 10 mM. HPG was then added to each of the crimped vials which when diluted with 75 mL of Ney fluids would achieve a final concentration of 100 μm HPG. Stock solutions of polysulfide, sulfide, thiosulfate, sulfate, and acetate were added anaerobically to the serum vials in small volumes of 3 mL or less so that they would achieve the following final concentrations once 75 mL of Ney Springs cistern fluids were added: 20 mM polysulfide, 20 mM polysulfide/10 mM acetate, 20 mM thiosulfate, 20 mM thiosulfate/acetate, 20 mM sulfide, and 20 mM sulfate/10 mM acetate. Serum vials were also prepared for background activity and fluorescence controls that only contained HPG or did not contain HPG or any other additives. In the field, 75 mL of Ney cistern fluid was added to each serum vial via syringe. Serum vials were all kept cold (~4°C–10°C) and in darkness before and after inoculation (~3 days). In the lab, the serum vials targeting sulfur oxidizers were then exposed to oxygen with a 0.1 μm syringe filter and needle through the septa, while sulfur reducing incubation were kept anaerobic. All incubations were allowed to incubate at room temperature for 3 weeks before they were filtered, as previous enrichment trials with sulfur species indicated this is a sufficient time to observe cell growth. Thiosulfate oxidizing control organisms, previously isolated from Ney springs (Trutschel et al. 2023), and *Shewanella oneidensis* were used as BONCAT positive label controls for this experiment.

For microscopy, 40 mL was removed from each serum vial and fixed with 3% PFA for 1 h. These were then filtered onto 0.1 μm polycarbonate membrane filters (47 mm diameter, Millipore, Tullagreen, Carrigtwohill Co. Cork, IRL). The filters were then rinsed with 10 mL 1:1 Ethanol:Phosphate buffered saline (PBS) and allowed to dry before storage at −20°C. Solutions for click chemistry were prepared according to the protocol, with AZdye 488 used as the azide dye and DAPI used as a counterstain. Stained filters were analyzed on a Nikon ECLIPSE TI‐E inverted microscope. Fifty images with a field of view measuring 0.18 mm × 0.18 mm were taken for each filter.

## 3 Results/Discussion

### 3.1 Ney Springs Cistern Fluids as a Source of Reduced Molecules and High pH Fluids to the Surrounding Area

High pH (12–12.7) and sulfidic (> 300 mg/L) fluids have been consistently measured at Ney Springs for more than 100 years (Waring 1915), with very little seasonal fluctuation observed (Trutschel et al. 2023). We have previously shown that this location is a major source of high pH connate fluids, enriched in reductants. Here we investigated the impacts of these fluids on the chemistry and microbiology of the surrounding systems (Figure 1 and Table 1).

**TABLE 1:** Representative samples of the aqueous geochemistry of Ney Springs cistern (N1), surrounding outflow (N2), and adjacent spring (N3) and creek environments.

	Ney cistern (N1)	Stairs outflow (N2)	Stagnant spring^1^ (N3)	Ney springs creek^1^ (Downstream)	Ney springs creek^1^ (Adjacent)	Ney springs creek^1^ (Upstream)
pH	12.5	12.2	10	9.2	8.356	8.35
Conductivity (μS/cm)	3830	3810	480	139	110	106
Dissolved oxygen (mg/L)	0.061	> 0.05^4^	> 0.05^4^	NA^2^	12.7	NA^2^
Sulfide (mg/L)	444	290	0.9	NA^2^	0.025	NA^2^
Thiosulfate (mg/L)	830	1050	< 0.05^3^	NA^2^	< 0.05^3^	NA^2^
Tetrathionate (mg/L)	400	< 0.05^3^	< 0.05^3^	NA^2^	< 0.05^3^	NA^2^
Sulfate (mg/L)	160	180	6.5	1.48	1.11	0.88
Chloride (mg/L)	4800	4500	420	7.24	0.56	< 0.02^3^

^1^ Stagnant Spring and Ney Springs Creek data is from October 2022 due to flooding in May 2023 creating inaccessible conditions.

^2^ “NA” indicates these values were not measured.

^3^ Indicates samples were below the detection limit of the method used (<).

^4^ Indicates a peak was detected via cyclic voltammogram scan using Au/Hg microelectrodes, which have a detection limit of 0.05 mg/L for dissolved oxygen (Luther et al. 2008).

Given the highly reduced nature of the cistern fluids, this environment is functionally anoxic, with only small amounts of oxygen being measured at the air‐water interface (Table 1). Fluids from the cistern slowly seeped into the surrounding area in measurable amounts resulting in distinct formations and biofilm communities. The stairs outflow interface environment (**N2**) provided an opportunity to investigate the impacts of the atmosphere and increased oxygen exposure on the Ney fluid chemistry and microbial community. Compared with the cistern pH (12–12.7), we consistently measured the stairs outflow (**N2**) fluids to be marginally lower in pH (Table 1). The water isotopes of the stairs outflow sample are the same as the cistern, with samples for both collected in October 2022 plotting on top of each other (Figure S1). In comparison, the nearby creek consistently measured slightly above neutral (pH 8.19–8.43), except for the area immediately downstream of the cistern and outflow fluids (within 3–5 m) which is notably more alkaline (pH 9.2) (Table 1). As stagnant spring (**N3**) was located very close to the creek bed, it is more impacted by surface/ground water interactions than the other locations and was occasionally inaccessible due to flooding. This is supported by the hydrogen and oxygen water isotopes, which suggest the spring fluids are largely influenced by meteoric water due to their close position to the global meteoric water line and distinction from the other Ney samples (i.e., **N3** vs. **N1** and **N2**; Figure S1). Nonetheless, the pH and sulfur concentrations at this spring point to stagnant spring providing an opportunity to compare the influence of fluid mixing between Ney's connate fluids with more oxidized meteoric fluids on microbial communities.

### 3.2 Minimal Isotopic Evidence for Biological Sources of Methane and Sulfide

As mentioned, the connate fluids at Ney Springs provide a source of reduced carbon, nitrogen, and sulfur species to the surrounding area; however, the origin of these molecules remains unclear. Methane is one of the most abundant carbon sources fluxing through the spring. It comprises approximately 80% of the exsolving gas which is consistently bubbling from the springs source in the main cistern. Timeseries analysis of methane isotopes in this study showed δ^2^H values consistently above −180‰ (−126.80‰ to −144.61‰) and enriched in ^13^C (δ^13^C of −16.5‰ to −19.62‰), both of which suggest a non‐microbial origin of methane (Liu 2019) (Figure S3). This was further supported by previously collected methane isotopologue data, from panorama (a high‐mass resolution multiple collector isotope ratio mass spectrometer) from collaborators for this system, which showed Ney Springs methane falling near the thermal equilibrium line at 50°C (Blank et al. 2017). While fractionation by methane oxidizing bacteria can cause enrichment in both ^13^C and ^2^H (Coleman 1981), preliminary clumped isotope work and the fact that previous work showed little evidence for either biological production or consumption of methane (Trutschel et al. 2022, 2023) make a geologic methane source the most parsimonious explanation. Additionally, the high ammonia concentrations at Ney Springs could be inhibitory towards methane metabolisms (Bedard and Knowles 1989; Gallert et al. 1998). The ammonia concentrations are anomalously high at this location compared with other serpentinizing systems, and previous work has suggested a potential biological origin of ammonia from Stickland reactions by the most abundant taxa in the spring (Trutschel et al. 2023).

The origin of the reduced sulfur species at Ney Springs remains unknown. Previous work has posited proximity to a volcanic system (Mt. Shasta), which could mean the Ney fluid chemistry is impacted by geothermal activity. However, the high nitrogen to argon ratio of exsolved gasses suggests there is limited geothermal influence (Mariner et al. 2003). Alternatively, the marine deposit that impacts the overall fluid conductivity and ion content in the spring could act as source for sulfate, which could be reduced microbially (Alt 2013). To investigate this potential, we collected sulfate and sulfide samples for isotopic analysis (Table S2). Interestingly, the sulfate and sulfide isotopic composition were relatively similar (δ^34^S_VCDT_ 14.34 vs. 16.67 and Δ^33^S or 0.024 and 0.056 for Zn‐S and sulfate samples respectively), suggesting that the sulfate molecules may result from sulfide oxidation (Alt 2013; Thode 1970). This is supported by our metagenomic work (discussed below) which demonstrated limited genetic evidence for sulfate reduction, but several taxa capable of sulfur oxidation in the Ney Springs cistern microbial community.

### 3.3 Oxygen and pH Impact Available Sulfur Forms at N2 and N3 Locations

**FIGURE 2:**
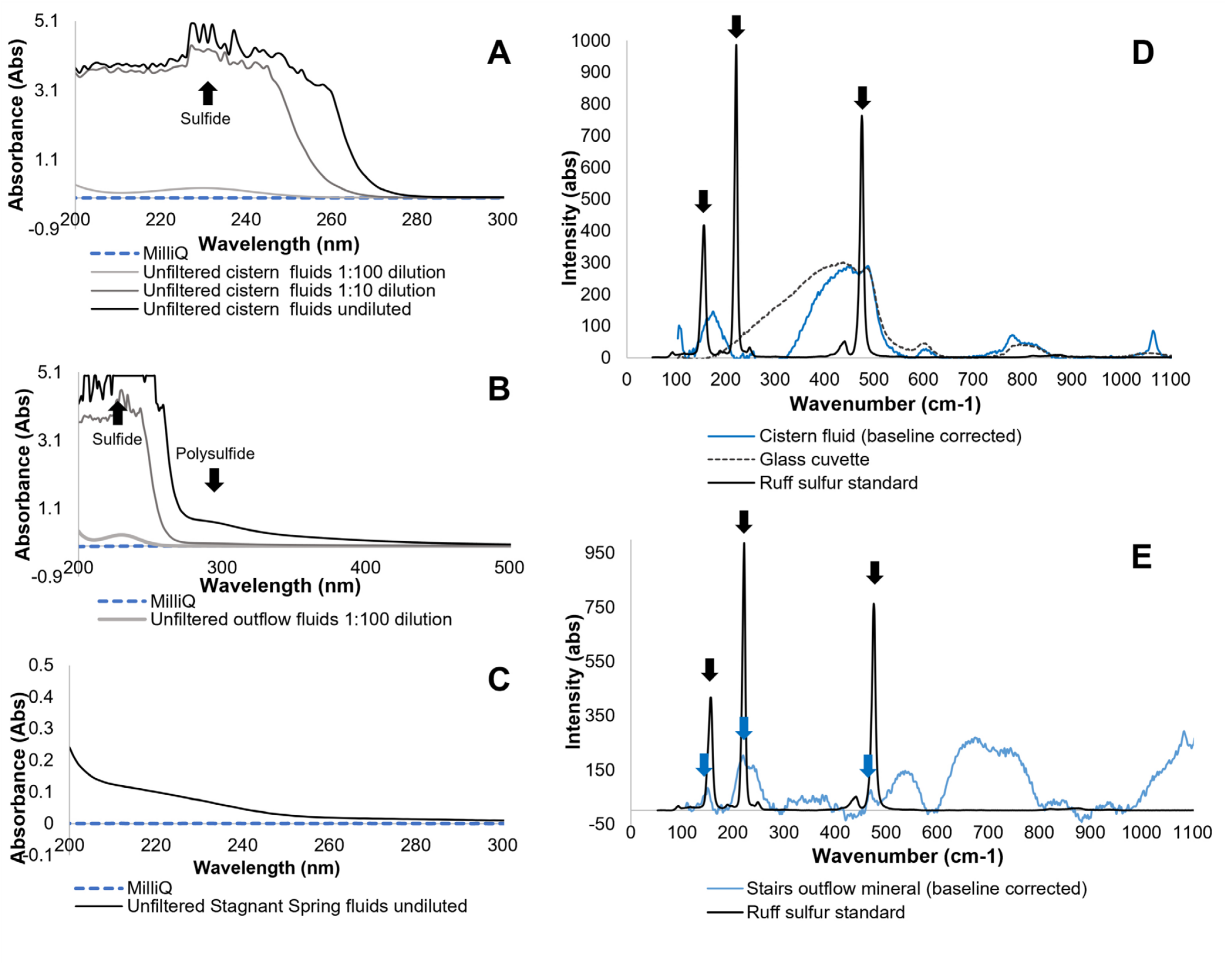
Identification of sulfide, and polysulfide in Ney Springs environments N1 (A), N2 (B) and N3 (C) using UV–Vis absorption spectroscopy. Raman spectroscopy of the cistern N1 (D) and stairs outflow environment N2 (E) compared to an elemental sulfur standard. The arrows represent key peaks found within the elemental standard, with matching peaks only observed in the N2 sample.

**FIGURE 3:**
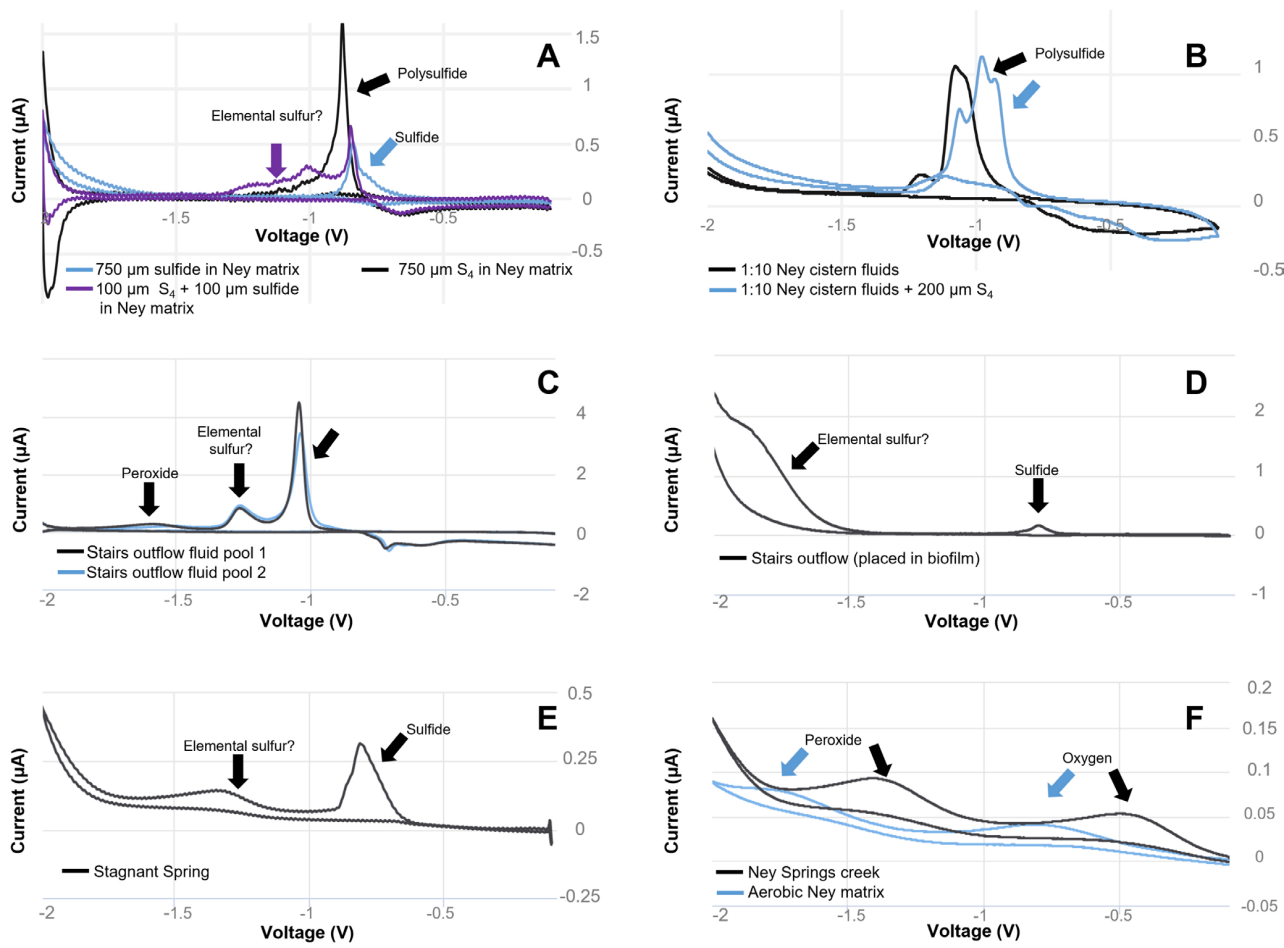
Geoelectrochemical data for: Standards of sulfide, polysulfide, and a mix of both substrates, run under cyclic voltammetry (X mV/s) in Ney Springs artificial medium, elemental sulfur predicted based on a previous experiment but noted as unconfirmed (?) due to the lack of a standard available (A); Ney cistern (N1) fluids diluted 1:10 in anoxic DI water, with and without a polysulfide standard addition (B); the stairs outflow environment (N2) in two separate locations (C and D) showing various geochemical characteristics; fluids observed at stagnant spring (N3) showing detectable sulfide and minimal oxygen; and Ney Creek showing oxygen only (peaks shifting due to pH).

**FIGURE 4:**
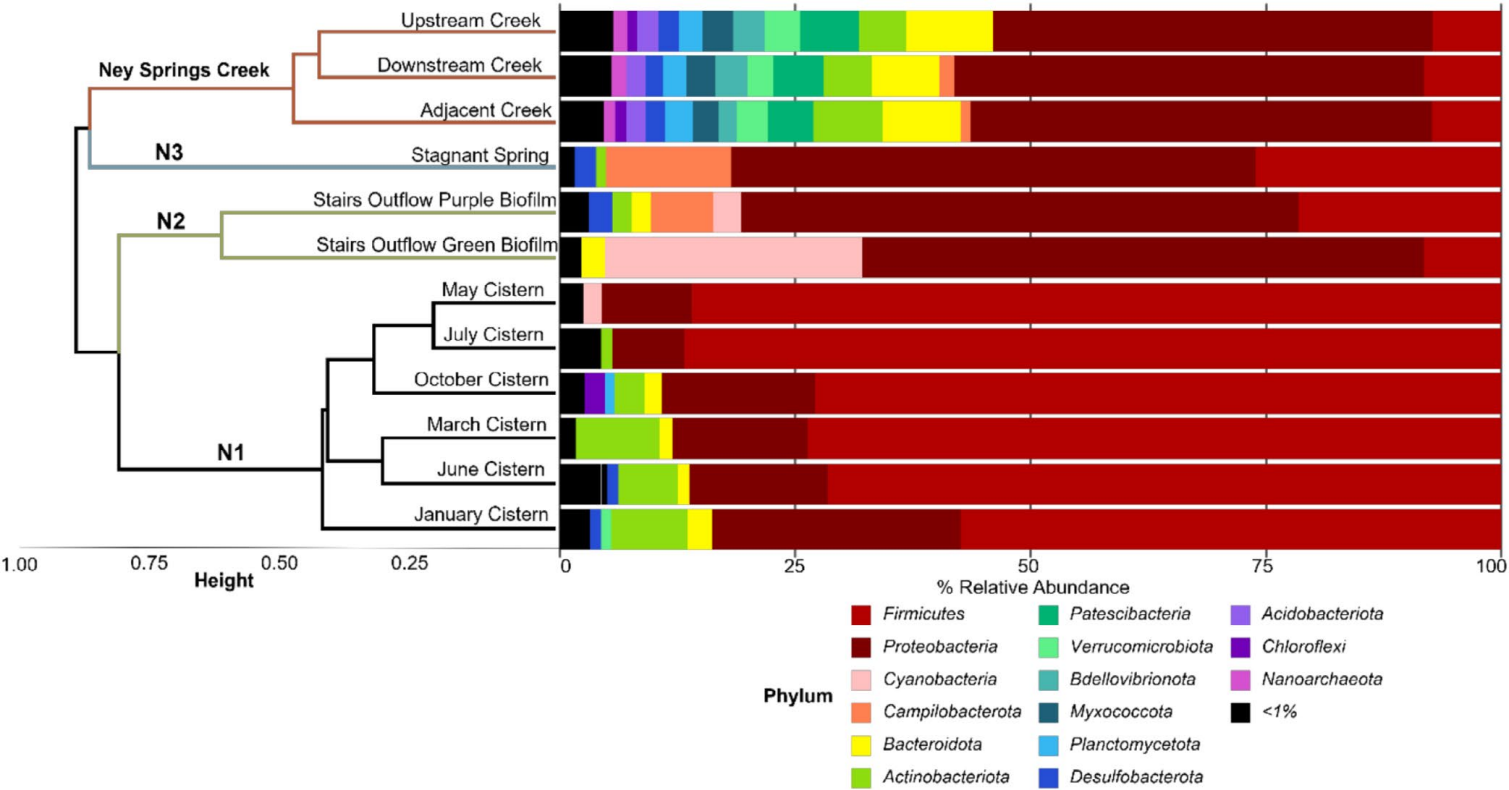
Dendrogram of Ney Springs samples calculated using Bray‐Curtis distance metrics. Sampling location in reference to map displayed in Figure 1 is noted in black on the appropriate branch. Taxonomic barchart of Ney Springs samples at the Phylum level. Samples are a merged average based on location, with the exception of the cistern samples which are additionally merged by month.

Oxygen availability plays a key role in the relative abundance of sulfur oxidizing taxa and the observed sulfur forms, throughout the Ney Springs system. The Ney cistern (**N1**) has a high concentration of sulfur species over a range of oxidation states. The highest concentration of sulfide (444 mg/L) was observed at **N1**, which is expected given that the cistern is largely anoxic and is primary output of the source fluids. The prevalence of sulfide is supported by Raman, UV–vis and geo‐electrochemical analyses (Figure 2). Interestingly the most abundant form of sulfur within the cistern is thiosulfate (830 mg/L), though other abundant oxidation sources are also present at lower concentrations (400 and 160 mg/L for tetrathionate and sulfate respectively) (Table 1). The complex nature of the various sulfur oxidation states observed within the cistern alone isexemplified by the detection of polysulfide and elemental sulfur. Although we are unable to quantify these sulfur species using cyclic voltammetry (CV), these data suggest a dynamic relationship between many of the sulfur intermediates observed within the Ney Springs cistern (Figure 3). Comparing the CV peaks formed with standards constructed in the anoxic Ney matrix (pH 12), sulfide and polysulfide were found to form peaks at close but distinct locations at −0.8 V and − 0.9 V when assessed separately but formed a triple peak when combined in solution together (Figure 3A). As spiking in known standards of sulfur species resulted in the formation of these triple peaks (Figure 3B), it was challenging to absolutely confirm the identity of the various peaks observed in the Ney Springs fluids. One of the potential explanations for these shifting and merging peaks is due to the presence of elemental sulfur in this system; elemental sulfur is likely present as both nanocrystalline and organically complexed elemental sulfur, which is electrochemically active and can interact with other species such as polysulfide and/or sulfide found within the cistern (Figure 3) (Boyd and Druschel 2013; Kafantaris 2017). Polysulfide is expected to be stable under these high pH conditions, and it is likely produced as a result of interactions between sulfide and the different elemental sulfur intermediates present. The Raman and UV–Vis spectra confirm some form of elemental sulfur is present in the Ney system as well (Figure 4), and in some samples these coincide with the form observed in our RUFF standard. Though we observe polysulfide electrochemically, and polysulfide should not be generated electrochemically as an artifact, we did not confirm polysulfide in the cistern fluids with an outside or lab‐based method (e.g., UV–vis or Raman). Given polysulfide oxidizes to thiosulfate and is more challenging to preserve, this is potentially why we only see it present with our electrochemical measurements, as these are performed in the field rather than collected as a sample and analyzed later. Nonetheless, these data support a wide range of redox states of available sulfur in the cistern, and we predict based on these observations and previous work (Trutschel et al. 2022, 2023) that oxygen exposure at the spring surface and microbial activity are responsible for these diverse sulfur intermediates.

Sulfur species composition shifts in the outflow systems, which have a notably larger surface area to fluid volume ratio compared to the main cistern. In the **N2** fluids, a decrease in sulfide (290 mg/L) and increase in thiosulfate (1050 mg/L) and sulfate (180 mg/L) was observed (Table 1). Interestingly, tetrathionate was below the limit of detection in the stairs outflow system, despite being consistently measured in the cistern. Higher oxidation state products, such as elemental sulfur, were detected in the Raman and geo‐electrochemical analyses of **N2** fluids and precipitates respectively (Figure 2 and Figure 3C,D). Polysulfide was also observed using geo‐electrochemical assays in some samples/locations and could be confirmed via UV–vis spectra (Figure 2 and Figure 3C,D). While the level of oxygen interaction may dictate which sulfur intermediates form and dominate each of the outflows, the dropping of pH and conductivity as the source fluids mix with meteoric water may additionally affect the generation and preservation of some of the observed sulfur species.

Unlike within the cistern, meteoric water mixes with other fluid flows in the Ney Springs area, which results in a lower fluid conductivity and overall lower sulfur concentration (Table 1). **N3** (stagnant spring) has the lowest detectable sulfide concentration but is still functionally anoxic with dissolved oxygen falling below the 0.05 mg/L detection limit (Table 1 and Figure 3E). Dilution of the connate fluids with meteoric water has reduced the total sulfur concentrations, however sulfide (up to 0.9 mg/L) is still consistently present in this system. Interestingly, the sulfate concentration (6.5 mg/L) is much higher than the total reduced species measured. Similarly, in the creek directly adjacent to Ney Springs, sulfide was detectable (0.025 mg/L) using the methylene blue assay, but at this more neutral pH and in the presence of oxygen (12.7 mg/L) this sulfide seems to quickly oxidize to sulfate (1.11 mg/L) which is more abundant in the downstream and outflow communities than in the upstream creek location (Table 1). Meanwhile thiosulfate and tetrathionate are not observed at detectable levels in either **N3** or within the creek locations sampled.

Combined, these data point to a diverse combination of sulfur intermediates present in the Ney Springs system. Overall, the observed differences in sulfur oxidation products between the cistern and stairs outflow environment is likely a combination of biotic and abiotic drivers such as abiotic interaction with oxygen, and/or the activity of sulfur oxidizing microorganisms. Quantification of elemental sulfur in such systems is extremely difficult to impossible (Kamyshny et al. 2004), and the techniques utilized here do not permit absolute quantification, but the presence of elemental sulfur and the interaction of that elemental sulfur and sulfide to form polysulfides (Avetisyan et al. 2019; Kafantaris and Druschel 2020; Kamyshny et al. 2004) is a key part of the observed chemistry. The high concentrations of thiosulfate are likely the result of polysulfide oxidation, as thiosulfate is the predominant product of this reaction (Kleinjan et al. 2005). Thiosulfate in turn would be susceptible to abiotic decomposition at these conditions, though in non‐acidic springs this would be relatively slow and tetrathionate is not typically a significant product of this process (Xu 1997; Xu et al. 2000). Given that significant tetrathionate is observed in **N1**, and that we have previously observed changes in tetrathionate concentration correlated with changes in abundance of putative tetrathionate producing genera such as *Halomonas* (Trutschel et al. 2023), future work will look to disentangle the role of biological sulfur oxidation in potentially enhancing this process. Notably, the presence of these intermediates at Ney Springs supports the fact that these products are at least moderately stable at extremely high pH.

The ratio of more reduced to oxidized forms of sulfide may place limitations on the sulfur oxidizing community in the cistern, as evidenced by the enrichment of sulfur‐associated taxa in the outflows compared to cistern as discussed in the next section. In the meteoric water influenced systems, the overall concentrations of sulfur species were lower, as they are driven by dilution with sulfur‐free fluids. However, the ratio of oxidized products increases from the pure connate fluids in the cistern as we move to the creek, thus supporting overall enhanced oxidation in the mixed fluids and greater overall abundance and variety in terminal electron acceptors available to the microbial community. It is not clear whether the shift in proportion of sulfur‐utilizing taxa at the interfaces is largely driven by enhanced oxygen exposure or due to enhanced availability of some other limiting nutrient (e.g., carbon dioxide), but these shifts in sulfur species presence and abundance can be further correlated with the physiologic potentials observed in several of the endemic microbial community members as discussed below.

### 3.4 Changes in the Microbial Community of the N2 and N3 Locations Compared to N1

Microbial community analysis via 16S amplicon sequencing across the Ney Springs system demonstrated distinct community profiles across the interfaces investigated. Unsurprisingly, the cistern community samples all group together, but are most closely related to the community observed in the stairs outflow **N2** (Figure 4)—the closest system. Stagnant spring, likely due to meteoric water influences, clusters more closely with the creek samples, and in particular the Ney Springs adjacent section (Figure 4). The overall alpha diversity of the microbial community is highest in the Ney Springs creek samples (avg. Shannon 7.08 ± 0.76, Avg. Simpson 0.99 ± 0.005), while the cistern (avg. Shannon 3.26 ± 0.84, Avg. Simpson 0.81 ± 0.06), stairs biofilm (avg. Shannon 2.92 ± 0.93, Avg. Simpson 0.85 ± 0.08), and stagnant spring samples (avg. Shannon 3.08 ± 0.08, Avg. Simpson 0.89 ± 0.008) share similar levels of diversity.

**FIGURE 5:**
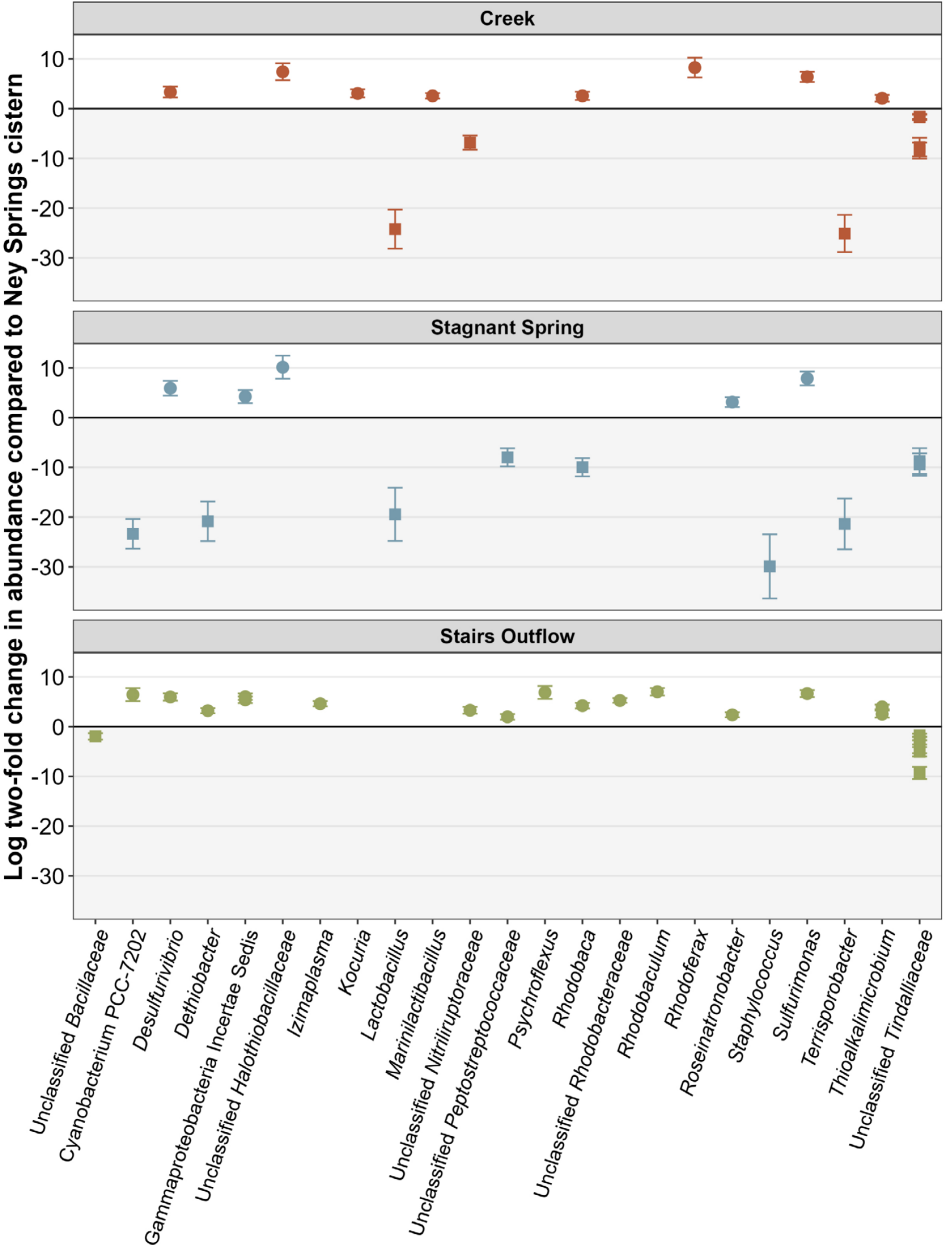
DEseq differential abundance plot of Ney Springs creek, Stagnant Spring (N3), and Stairs Outflow (N2) microbial communities compared to the Ney Springs Cistern (N1).

As the **N1** and **N2** communities are proximal, and fluids from **N1** flow to **N2**, observed shifts in the microbial community between these two systems are likely a result of the environmental and geochemical differences. Besides the introduction of new taxa from the mingling of source fluids with the surrounding neutral environment at these interfaces, many of the taxa regularly found within **N1** are significantly enriched at these other locations (Figure 5). As previously described, the cistern microbial community is dominated by fermentative firmicutes belonging to the *Tindallia* and *Izimaplasma* genera. These genera consistently make up the majority of the community, with anywhere from 50% to 80% of the community comprised of a few species belonging to these two groups (Trutschel et al. 2022, 2023). Though abundant within the cistern, *Izimaplasma* is notably more enriched within the **N2** biofilm compared to the cistern (Figure 5). Biofilm growth has not been observed within the cistern, while rich biofilm communities are a predominant feature of the stairs outflow, which also likely impacts community composition. Mixing in the cistern may select against biofilm formation, while the constant flow of fluids in the outflows favors this ecology. The stairs outflow biofilm is also more sun exposed than the cistern and thus is predominately composed of Proteobacteria and Cyanobacteria, with *Cyanobacterium* PCC‐7202 and various potentially phototrophic *Rhodobacteraceae* spp. comprising the most abundant taxa (Data S1). Additionally, many of the putative sulfur‐oxidizing taxa observed within the cistern fluids are more enriched within the stairs outflow samples as well, including several *Rhodobacteraceae* genera, *Sulfurimonas*, and *Thioalkalimicrobium* (Figure 5). The enrichment of these putative sulfur‐oxidizing taxa within **N2** may be explained by the increased availability of both oxygen as a terminal electron acceptor and the availability of multiple sulfur sources in the forms of thiosulfate, sulfide, elemental sulfur, and polysulfide.

Though the overall community of stagnant spring is more similar to creek samples, rich biofilms with streaming communities can be observed at **N3** that were not seen in the creek (Figure 1F). Similarly, like the stairs outflow community, **N3** is enriched in more putative sulfur‐utilizing taxa compared to the cistern fluids, and includes higher amounts of *Desulfurivibrio*, *Roseinatronobacter*, *Sulfurimonas*, and a *Halothiobacillaceae* species (Figure 5). The *Halothiobacillaceae* species is the most abundant ASV in the stagnant spring biofilm, making up approximately 30% of the community (Data S1). *Halothiobacillaceae* spp. are in low abundance within the cistern fluids and other outflow areas but seem to flourish in stagnant spring, suggesting these species may be less tolerant of extremely high pH, but still benefit from the presence of several sulfur species.

Both upstream and downstream Ney Springs creek locations show a similar microbial community composition, even though sample were taken 75 m apart. Many of the most abundant ASVs found in the cistern, such as *Tindallia*, *Izimaplasma*, and various *Rhodobacteraceae* were also present in the creek samples. The creek samples were moderately enriched in *Rhodoferax, Sulfurimonas* and *unclassified Halothiobacillaceae* compared to the cistern (Figure 5). While many of the most abundant taxa within the creek are also found in the cistern, two genera stand out as more distinct elements of the creek environment: *Rhodoferax* and *Thiothrix*. While found in low numbers throughout locations around Ney Springs, *Rhodoferax* is the first or second most abundant ASV within all sampled creek locations. The creek also contained high amounts *Thiothrix*, a common sulfur‐oxidizing taxa, which was absent from most other locations except stagnant spring.

### 3.5 Light, Oxygen, pH and Sulfur Physiologies Correlate With Community Changes in the N2 and N3 Communities

**FIGURE 6:**
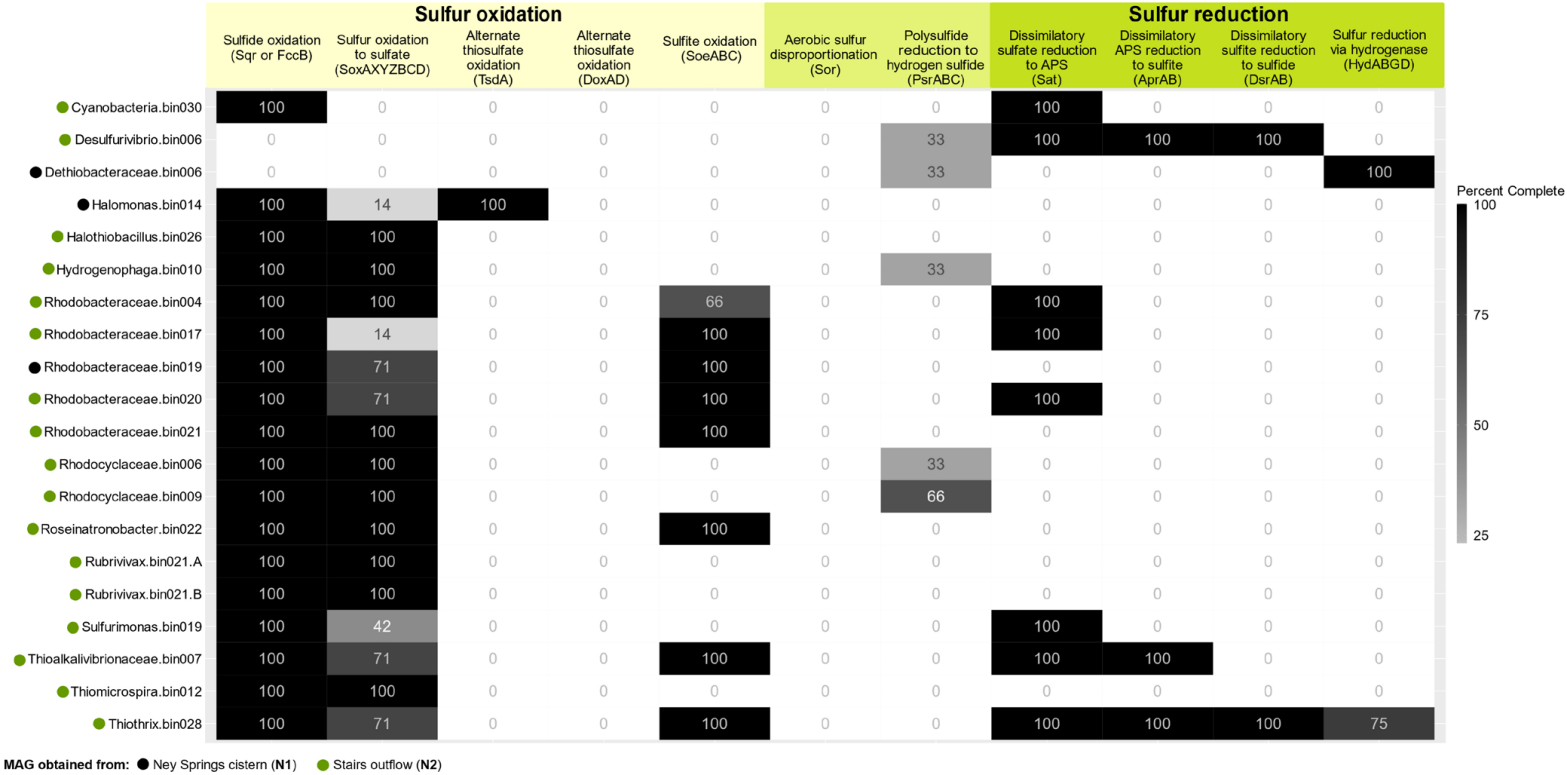
Heatmap of sulfur metabolism genes found in metagenome assembled genomes (MAGs) obtained from the Ney Springs system. *Key indicates sample location where the MAG originated from*.

In addition to increased exposure to oxygen, the outflow communities are more exposed to light, and this has likely impacted the relative abundance of photosynthetic taxa. Enrichment of *Rhodobacteraceae* and *Cyanobacterium* groups (putatively capable of phototrophy) within the stairs outflow biofilm is seen, which are likely to correspond with the purple and green pigments often observed in the biofilm. The enhanced accessibility of sunlight in this area compared to the cistern may explain why these genera are more enriched in the stairs outflow region compared to the cistern (Figure 5). *Rhodobacteraceae* species such as *Rhodobaculum* are important core community members of the Ney Springs cistern, but no pigmented cultured representatives have been observed or isolated from this system thus far (Trutschel et al. 2022, 2023). The type strain of *Rhodobaculum* was isolated from a pH 9 soda lake and is described as an alkaliphilic photoheterotroph that forms pale‐pink to dark purple colonies (Bryantseva et al. 2015). Although the type strain of *Rhodobaculum* was not shown to oxidize any sulfur species, it is a part of the *Rhodobaca*‐*Roseinatronobacter* alkaliphilic *Rhodobacteraceae* clade, which has varying degrees of photosynthetic and sulfur‐oxidizing capabilities (Bryantseva et al. 2015; Kopejtka et al. 2018, 2017). Several MAGs belonging to the *Rhodobacteraceae* have been pulled from both the cistern (**N1**) and stairs outflow (**N2**) locations (Figure 6). Notable traces of cyanobacteria found within the system are 16S sequences belonging to *Cyanobacterium* PCC‐7202 (*Synechococcus*) species, the type strain of which was originally isolated from an alkaline lake in Chad and is said to be adapted to saline and freshwater conditions (Klanchui et al. 2017). Additionally, a *Cyanobacterium* MAG identified as a *Pleurocapsa* species was also obtained from a stairs outflow sample (Table S1).

As previously mentioned, the diversity and distribution of sulfur intermediates was linked to oxygen exposure and/or the introduction of meteoric water. For example, abiotic oxidation of sulfide and/or polysulfide can result in the production of thiosulfate, and this molecule is enriched in the stairs outflow system (**N2**) as are many members of the *Rhodobacteraceae*. These organisms are capable of thiosulfate oxidation, and both metagenomic and cultured isolate evidence of thiosulfate oxidation has been shown for members of this family obtained from Ney Springs (Figure 6) (Trutschel et al. 2022; Trutschel et al. 2023). MAGs belonging to members of this family all encode for sulfide:quinone oxidoreductase (Sqr), sulfide dehydrogenase flavocytochrome (FccB), and may also contain a complete or partial Sox pathway, sulfite: quinone dehydrogenase (SoeABC) and/or sulfate adenylyltransferase (Sat) (Figure 6) supporting the capacity for sulfur oxidation within members of this group, though these organisms are also confirmed heterotrophs. Conversely, *Thiomicrospira* (a.k.a. *Thioalkalimicrobium* or *Hydrogenovibrio*) and *Sulfurimonas* are putative autotrophic sulfur oxidizers enriched at this location. *Thiomicrospira* has previously been established as an abundant cistern core community member (Trutschel et al. 2023) and has been previously detected in marine serpentinizing systems (Brazelton et al. 2012; Postec et al. 2015). *Sulfurimonas* species are known for their ability to colonize a wide range of environments and utilize both hydrogen and sulfur as electron donors (Han and Perner 2015). Both *Thiomicrospira* and *Sulfurimonas* are predicted to utilize Sqr and/or FccB for oxidation of sulfide along with the Sox system for oxidation of thiosulfate (Han and Perner 2015; Scott et al. 2006), which is consistent with current genomic observations, though the MAG obtained for *Sulfurimonas* (predicted to be 97% complete with < 2% contamination; Table S1) only contains Sox YCD. Species from both genera are often described as obligately autotrophic, using the energy gained from sulfur oxidation to fix carbon (Han and Perner 2015; Scott et al. 2006; Sorokin et al. 2002). In addition to enhanced oxygen exposure in the overflow environment due to an increase in the surface area to volume ratio for atmospheric exposure, there is also potential for greater access to bioavailable inorganic carbon from the atmosphere (e.g., carbon dioxide) in the stairs outflow environment. This may aid the aforementioned putative autotrophs with growth and carbon fixation in the outflow environment, as inorganic carbon is notoriously limited at high pH due to precipitation of carbonate with divalent cations to form solid species such as calcite (Schrenk et al. 2013).

Though thiosulfate is higher in the stairs outflow fluids, the sulfur oxidation intermediate tetrathionate was only measured within the cistern. Long term seasonal sampling of the Ney Springs cistern revealed a positive correlation between *Halomonas* ASVs and increasing tetrathionate concentrations (Trutschel et al. 2023). A *Halomonas* sp. also remains the only MAG pulled out of the system with a putative alternate thiosulfate oxidase (DoxAD or TsdA) that would theoretically produce tetrathionate as an intermediate (Figure 6). Additionally, a *Halomonas* isolate from the cistern has been shown to produce tetrathionate as a sulfur oxidation intermediate (Trutschel et al. 2022). Notably however, *Halomonas* is not significantly enriched in the cistern compared to any of the other locations and can be found in abundance throughout all locations sampled (Data S1). As such, *Halomonas* may be metabolically active throughout the system via heterotrophy, but either the anoxic nature of the cistern better preserves the tetrathionate, or the cistern environment allows them to better compete for thiosulfate.

In addition to providing favorable conditions for aerobic sulfur oxidizers, the presence of multiple sulfur species such as sulfide, thiosulfate, elemental sulfur, and sulfate may also generate more favorable conditions for organisms that utilize sulfur disproportionation and consequently we see more evidence for these metabolisms in the MAGs associated with taxa that are enriched in **N2** compared to **N1**. *Desulfurivibrio* and *Dethiobacter* are putative sulfur‐utilizing taxa that have been encountered at Ney Springs previously, but not in regular abundance within the cistern (Trutschel et al. 2023). A *Desulfurivibrio* MAG obtained from the cistern contains a complete set of genes necessary for sulfate reduction (Figure 6) (Trutschel et al. 2022), however the only isolate for this genus, *Desulfurivibrio alkaliphilus* , grows as a sulfur oxidizer or sulfur disproportionator in culture (Melton et al. 2016; Thorup et al. 2017). Similarly, *Dethiobacter* isolates from soda lakes have also been shown to engage in sulfur disproportionation using polysulfide, thiosulfate and elemental sulfur but lack the ability to reduce sulfate (Merkel et al. 2023; Sorokin et al. 2008). The stairs outflow biofilm was the only location to contain detectable levels of the three sulfur intermediates: thiosulfate, elemental sulfur, and polysulfide.

The **N3** system is characterized by a lower pH relative to the other serpentinized fluid flows, and more dilute sulfur due to meteoric water dilution. The most abundant *Halothiobacillaceae* ASV enriched there is most closely related to *Thiofaba tepidiphila* , a chemolithoautotrophic sulfur‐oxidizing bacterium isolated from a hot spring (Mori and Suzuki 2008). Organisms from this family are typically halotolerant and often found in neutral or acidic environments, while its sister family *Thioalkalibacteraceae* is more strongly associated with haloalkaline environments. A *Halothiobacillus* MAG obtained from the system contains homologous duplicates of Sqr and FccB along with a complete Sox system (Figure 6), suggesting it is capable of both sulfide and thiosulfate oxidation.

The creek community, despite being fresh water, is enriched in a few sulfur oxidizing ASVs (Figure 6) such as *Rhodoferax* spp. These taxa have been detected in many aquatic environments, with many isolates displaying diverse metabolic capabilities related to sulfur oxidation, phototrophy, and even iron reduction (Jin et al. 2020). This ASV is observed in all the Ney samples but is enriched in the creek relative to the cistern. *Thiothrix* on the other hand is only abundant in the downstream creek location, where long hair‐like strands, characteristic of this filamentous organism, are visible on rocks within the creek bed (Figure 1G). *Thiothrix* are common in sulfide‐rich waters, and have been observed in areas impacted by mine drainage and wastewater (Larkin and Shinabarger 1983; Ravin et al. 2021). While the presence of *Thiothrix* immediately below Ney Springs best exemplifies the impact of Ney Springs fluids on the creek environment, the slightly elevated pH (pH 8.35) and the presence of other cistern‐associated taxa upstream of the cistern suggests there may be other access points for serpentinized fluids into creek, though this has yet to be established.

### 3.6 Sulfur Intermediates Are Likely the Dominant Substrate for Microbial Sulfur Oxidation in the Ney Springs System

**FIGURE 7:**
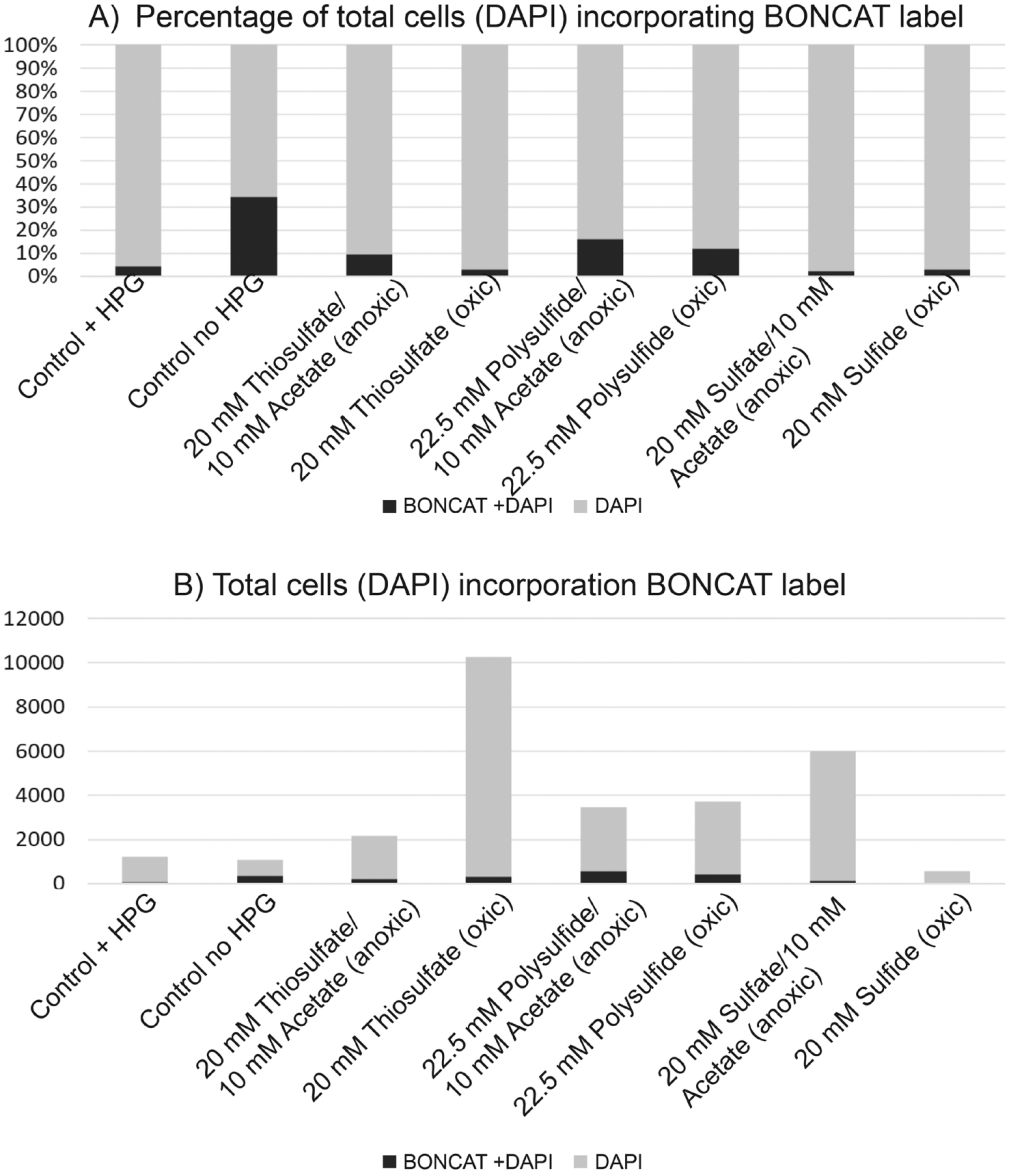
BONCAT incorporation for all DAPI labeled cells quantified in Ney springs incubations including control samples lacking any substrate addition, and those where electron donor and acceptors were provided. Experiments were performed aerobically unless otherwise stated. (A) Percentage of BONCAT and DAPI labeled cells relative to just DAPI labeled cells. (B) Total labeled BONCAT and DAPI labeled cells compared to DAPI only labeled cells.

In our previous work, we successfully isolated sulfur‐oxidizing organisms relevant to the Ney Springs environment, including *Thiomicrospira* sp. (Trutschel, unpublished), a *Halomonas* sp., and a *Rhodobacterceae* sp. (Trutschel et al. 2022). Interestingly, most of these successful cultivars came from inoculations using polysulfide as an electron donor sulfur substrate, rather than sulfide. To confirm our hypotheses about the metabolic potential of organisms with different sulfur species in this system more broadly, we attempted to use a BONCAT labeling approach (Hatzenpichler and Orphan 2015). BONCAT utilizes the uptake of HPG (an analog amino acid of methionine) and click chemistry to track metabolic activity under various experimental conditions. In this instance, HPG was spiked into several different incubations targeting various sulfur oxidation or reduction metabolisms to determine which of these conditions generated the most metabolic activity. Surprisingly, we noticed that no incubation had a larger percentage of HPG incorporated than the control samples, even though the total number of cells for certain incubations increased over five‐fold (Figure 7A). For example, cell numbers for the thiosulfate and polysulfide oxidizing incubations increased from approximately 1000 cells in the no growth addition experiments to 10,000 and 4000 cells quantified respectively. The acetate‐containing incubations also increased in the rage of 2000 to 6000 cells depending on the incubation (Figure 7B). This supported that while overall cell numbers were increasing in these incubations, we did not see a corresponding increase in fluorescence, suggesting that in this system, HPG was not incorporated into the growing population. Interestingly, our incubation with sulfide, the highest energy sulfur species for oxidation, yielded neither a significant increase in biomass nor BONCAT label incorporation, supporting that sulfide is not actively used by the microbial community in this high pH system. This is consistent with previous observations that our isolated representatives are incapable of growth in the presence of sulfide. Potentially, the bioavailability of sulfide at this high pH may be impacted as at a pH ~12, the primary sulfide species is S^2−^, which has decreased membrane permeability in its charged state (Sorokin et al. 2011; Steudel 2020). Further experimentation with isolates that can utilize multiple sulfur sources and grow across a broad pH range may help in addressing this question.

The general lack of labeling for BONCAT in our samples, including in samples that demonstrated increases in overall biomass, suggests that the microbes at Ney Springs may not universally take up the HPG label. To test this hypothesis, we performed BONCAT on the *Rhodobacteraceae* isolated in our previous work (Trutschel et al. 2022) growing aerobically on acetate in the presence of HPG. At the end of the growth curve where the OD increased from 0.05 to 0.4, we did not observe significant fluorescence signal in the cells, whereas using another lab strain, *Shewanella oneidensis* MR‐1 as a positive control we were able to observe label incorporation (Figure S4). While not an ideal result, this observation presents a potential caution for the application of BONCAT in highly divergent systems.

## 4 Conclusions

Throughout the Ney Springs systems, gradients of pH, oxygen concentrations, light and CO_2_ availability, as well as a diverse array of sulfur species can be observed stemming from various interactions with the Ney Springs connate fluids. These conditions generate a suite of distinct niches for microbial growth, and we have shown there are subsequent shifts in microbial community composition and structure, particularly with respect to various sulfur‐utilizing capabilities. For example, the stairs outflow environment results in robust biofilms that are enriched in taxa associated with autotrophic microbes including sulfur‐oxidizing *Thiomicrospira* and *Sulfurimonas* taxa or phototrophic *Rhodobacteraceae* and *Cyanobacterium* groups. This environment has the same source fluids as the cistern, but the enhanced exposure to light and the atmosphere provides more oxygen and access to CO_2_ for aerobic autotrophs. More broadly, we see the presence of sulfur‐oxidizing taxa, in sites that geochemically we would not expect (i.e., the oxic creek) due to the consistent influence of these fluids. The effects of this can be seen with the enrichments of members belonging to the *Halothiobacillaceae* within stagnant spring, and *Thiothrix* within the Ney Springs creek.

*Thiomicrospira*, *Halomonas*, and members of the *Rhodobacteraceae* have previously been associated within alkaline environments (Brazelton et al. 2006; Kopejtka et al. 2017; Sorokin 2003), but the presence of *Sulfurimonas, Thiothrix*, and *Halothiobacillaceae* members in an alkaline environment and/or in areas impacted by alkaline fluids offers new insight into the range of pH tolerance for these organisms. While isolation and enrichment attempts for many of the abundant core cistern community members have been unsuccessful, likely due to the low biomass observed in fluids, biofilm growth observed within the outflow and stagnant spring communities may aid in future cultivation attempts for organisms such as *Izimaplasma*, *Sulfurimonas*, *Halothiobacillus*, and additional members of the *Rhodobacteraceae*, as it provides a more concentrated source of biomass. This approach has already been proven successful with *Thiomicrospira*, as a few strains were recently isolated from the stairs outflow biofilm on solid media and have been successfully grown in vitro autotrophically with thiosulfate or polysulfide as the sole energy source (Trutschel, *unpublished*). *Thioalkalimicrobium/Thiomicrospira* forms a large portion of the Ney Springs gradient community, but it is also found in other serpentinizing systems such as the Lost City and Prony Bay hydrothermal fields (Brazelton et al. 2006; Postec et al. 2015), making it a key sulfur‐oxidizer across serpentinizing systems. Further isolate characterization and genomic investigation of this organism, as well as other dominant organisms from the Ney Springs gradient community, will improve our understanding of sulfur use across a wide pH range and what the genetic underpinnings to these processes may be.

Additionally, this work also provides an example of a system where BONCAT labeling did not faithfully identify anabolic activity in the microbes present. As this was not the focus of our study, we did not extensively troubleshoot these experiments nor exhaustively test this approach, but this may serve as the impetus for follow‐up work. Future work will investigate the origin of the challenges we observed in BONCAT in this system, including the cause of these labeling issues and whether they are specific to high pH conditions or are an organism‐specific phenomenon.

## Conflicts of Interest

The authors declare no conflicts of interest.

## Supporting information


Data S1.

Data S2.

Data S3.

## Data Availability

The data that support the findings of this study are openly available in NCBI at https://www.ncbi.nlm.nih.gov/, reference number PRJNA739719.
