# Supplementary material for: Sulfide Oxidation Products Support Microbial Metabolism at Interface Environments in a Marine‐Like Serpentinizing Spring in Northern California
Source: Geobiology. 2025 Jun 25;23(4):e70026. doi: 10.1111/gbi.70026 (PMC12198100; doi:10.1111/gbi.70026)
Supplement: Supplementary file 3 — Data S3. [file GBI-23-e70026-s002.docx]

Supplemental Figures and Data for:

**Sulfide oxidation products support microbial metabolism at interface environments in a marine-like serpentinizing spring in Northern California**


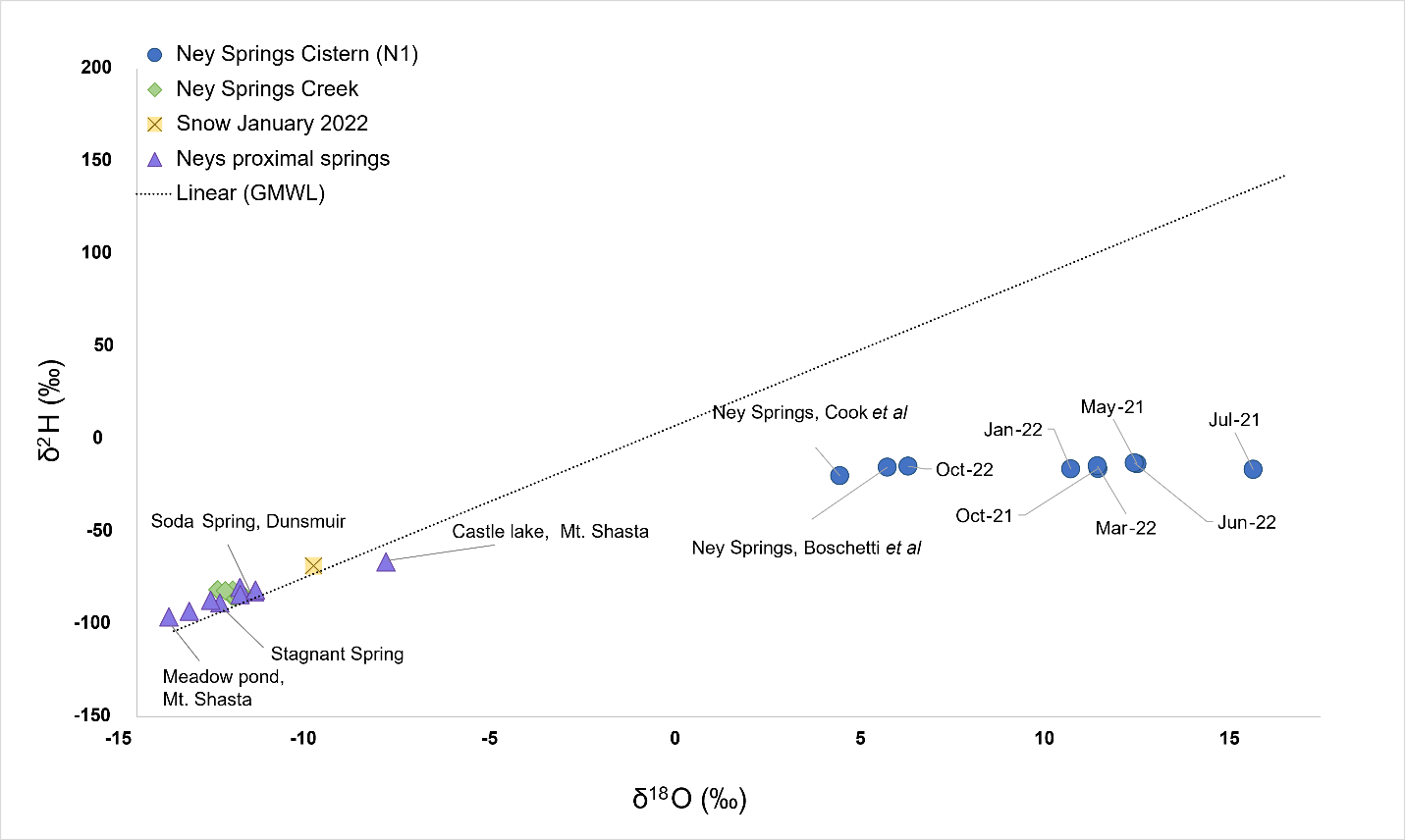


Supplemental Figure 1

Water isotope values for Ney Springs cistern, Ney Springs Creek, snow melt, and surrounding water sources around the Mt. Shasta-Dunsmuir area. Each point is based on a single water sample with δ^2^H sample precision of ± 0.6^0^/_00_ and a δ^18^O sample precision of ± 0.12^0^/_00_.


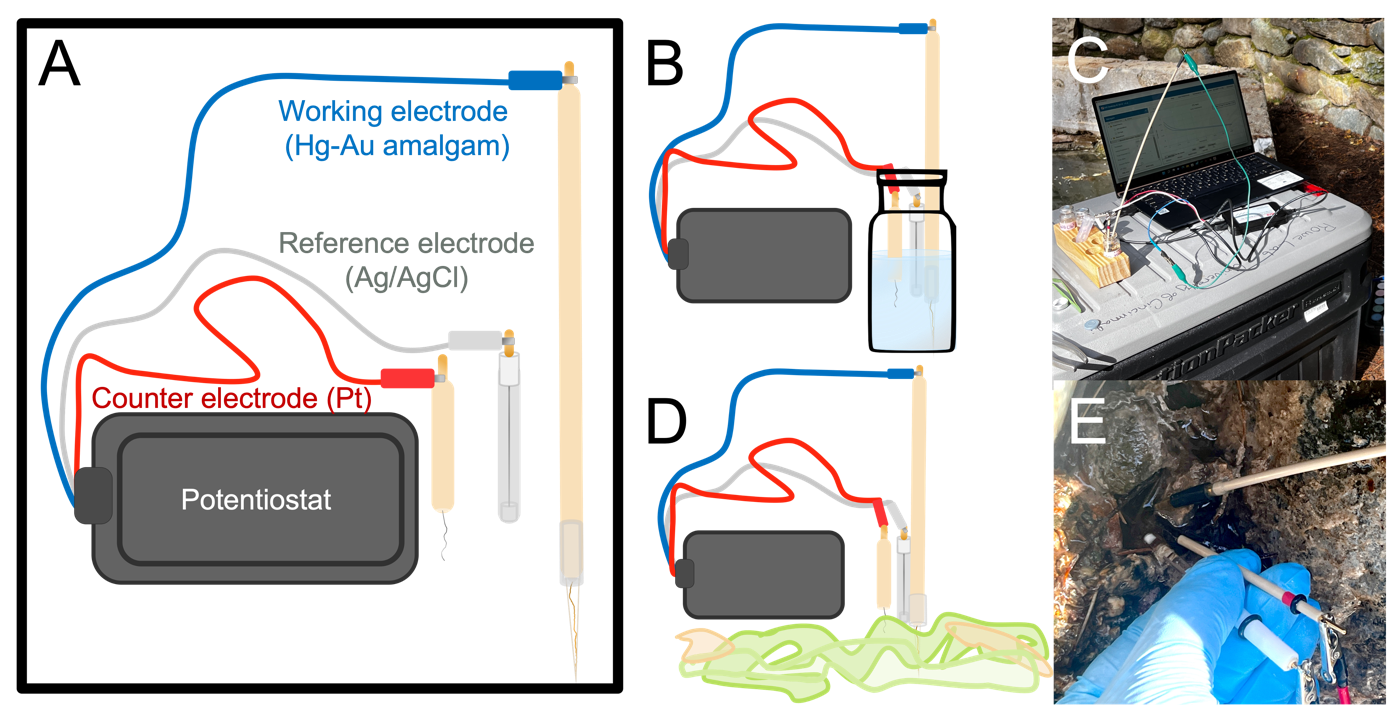


Supplemental Figure 2

((A) Diagram of AIS potentiostat and 3-electrode set up. (B) Diagram of setup when testing fluids in vials. (C) Photograph of testing cistern fluids in vials in the field. (D) Diagram of setup when testing biofilm. (E) Photograph of electrodes while testing outflow fluids in situ.


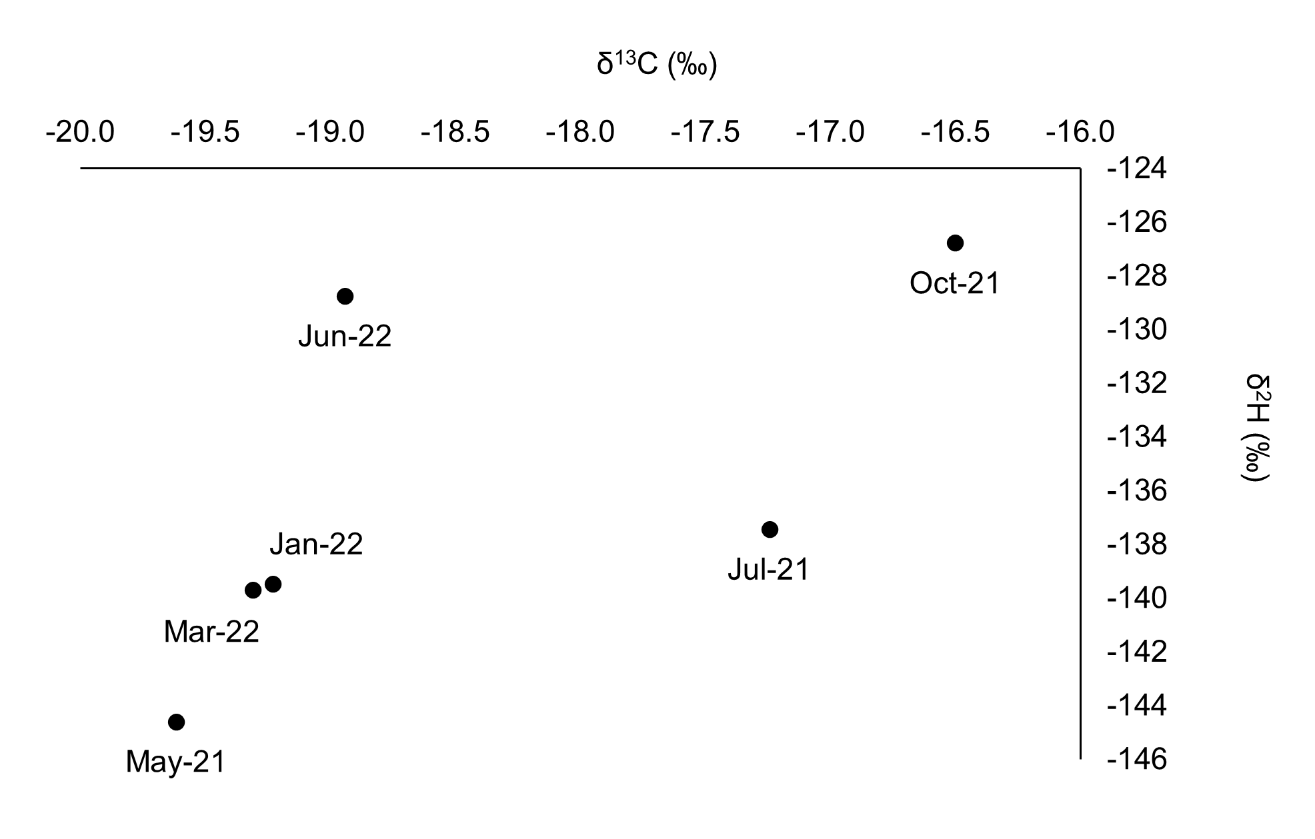


Supplemental Figure 3

Isotope measurements for exsolved methane gases collected at Ney springs from 2022-2023 performed at the UCD-SIF as discussed in Materials and Methods. The long-term standard deviation of these values reported are ± 0.2^0^/_00_ and ± 2^0^/_00_ for δ^13^C-CH_4_ and δ^2^H-CH_4_ respectively.


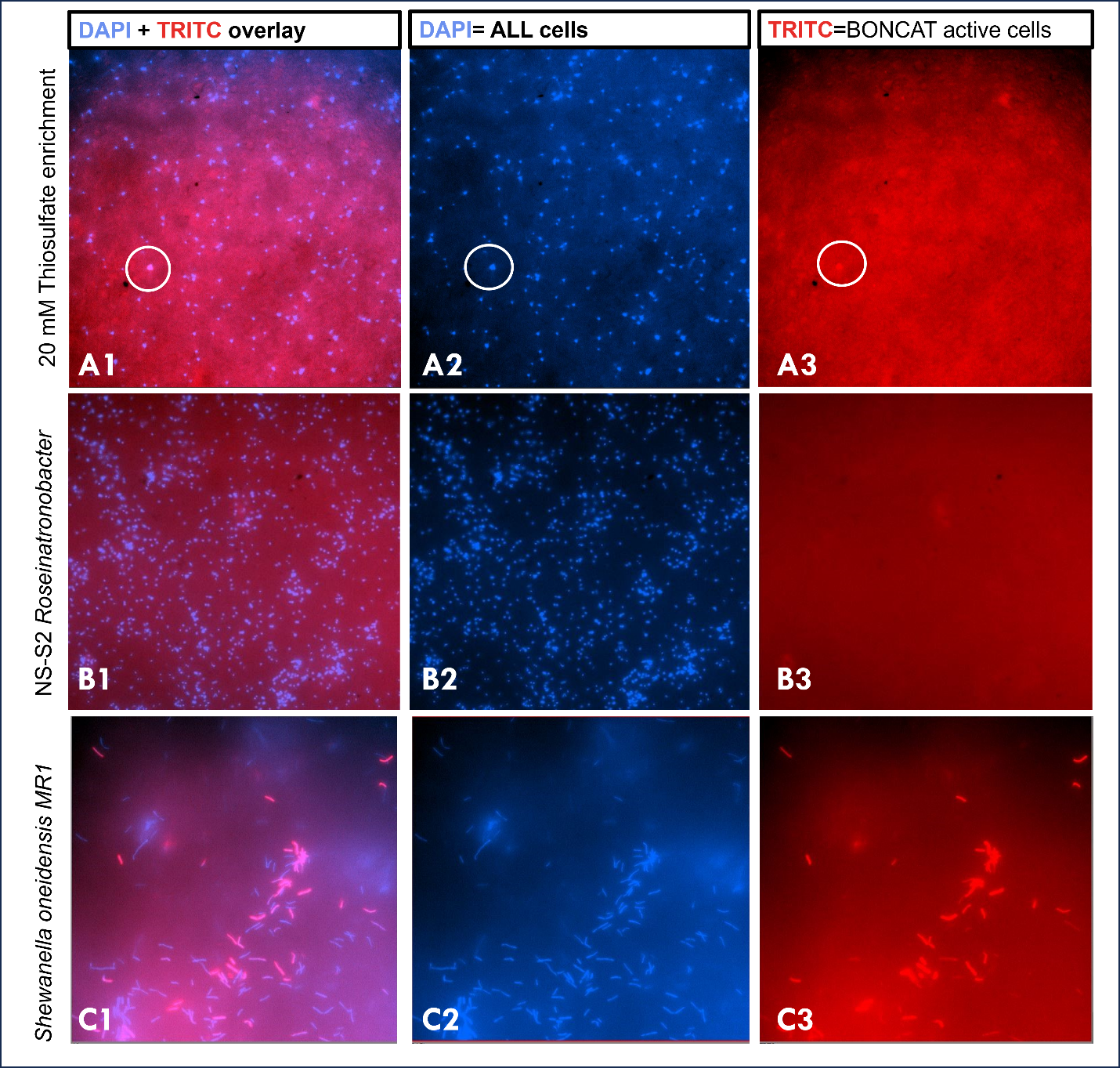
Supplemental Figure 4

Fluorescence micrographs of cells from Ney springs (row A), or control cultures of Roseinatronobacter NS-S2 (Row B) and Shewanella oneidensis MR-1 (Row C) stained for incorporation of a non-canonical amino acid (HPG) and treated with a fluorescent tag and click chemistry, (column 1&3) or with DAPI to stain nucleic acids (Column 1&2).

Supplemental data 1

Excel spreadsheet of taxonomic classifications and counts for all ASVs detected within the Ney Springs samples.

Supplemental table 1

Attached Excel File with MAG statistics and full classifications

*Supplemental table 2:* Isotopic measurements for sulfide and sulfate analyzed from Ney Springs taken at the same time as IAEA-S1 standard. Samples prepared as discussed in the methods by MIT Geobiology lab with one standard deviation for each sample reported.

| **Sample** | **δ^34^S_VCDT_** | **σ** | **Δ^33^S** | **σ** | **Δ^36^S** | **σ** |
| --- | --- | --- | --- | --- | --- | --- |
| IAEA-S1 | -0.32 | 0.03 | 0.082 | 0.012 | -1.07 | 0.070 |
| Sulfide (ZnS) | 14.16 | 0.00 | 0.024 | 0.020 | 0.25 | 0.051 |
| Sulfate (Thode) | 16.67 | 0.01 | 0.056 | 0.004 | 0.00 | 0.033 |
